# Statins in Membrane Lipid Therapy: Mechanistic Promise Versus Clinical Reality

**DOI:** 10.3390/ijms27125303

**Published:** 2026-06-11

**Authors:** Giulio Preta

**Affiliations:** Institute of Biochemistry, Life Sciences Center, Vilnius University, Saulėtekio av. 7, LT-10257 Vilnius, Lithuania; giulio.preta@bchi.vu.lt

**Keywords:** statins, cholesterol, pleiotropic effects, membrane lipid therapy

## Abstract

Statins are widely used to inhibit cholesterol synthesis and help prevent cardiovascular diseases associated with elevated blood cholesterol levels. Several clinical trials have shown that some of the benefits observed with statins are independent of cholesterol lowering. Part of these pleiotropic effects is related to the ability of statins to directly interact with and alter the physical properties of lipid membranes such as lipid packing and, consequently, membrane fluidity. Membrane lipid therapy is an innovative approach based on the use of drugs with the potential to change membrane lipid structure for therapeutic purposes, including enhancing the intracellular delivery of chemotherapeutic drugs. Evidence from studies using both artificial model membranes and biological membranes supports the potential use of statins as effective drug candidates for membrane lipid therapy. However, the transition from the promising *in vitro* results to the clinical practice has encountered significant challenges. This commentary highlights the pitfalls in repurposing statins as membrane-modifying agents.

## 1. Introduction

Drug repurposing is the practice of identifying new therapeutic uses for existing drugs or drug candidates beyond their original medical indication [1]. A classic example of drug repurposing is Sildenafil, originally developed to treat angina but later repurposed and approved as Viagra for treating erectile dysfunction. An ideal candidate for drug repurposing is a compound that has already progressed through clinical development and has well-established safety and pharmacokinetic profiles, with little to no observed toxicity. From this perspective, statins, widely regarded as well-tolerated and highly effective cholesterol-lowering agents, represent an excellent option for repurposing. Over the years, numerous studies have demonstrated that statins exert pleiotropic effects beyond their lipid-lowering function, with their potential anti-tumor properties drawing particular interest [2]. The rationale for using statins in the treatment of malignant diseases, often in combination with chemotherapy, includes their ability to promote apoptosis, regulate the cell cycle, and modulate epigenetic processes such as DNA methylation [3,4]. In this context, lipophilic statins are considered particularly promising candidates for clinical trials due to their enhanced ability to penetrate the lipid bilayer, greater cellular uptake, and broader tissue distribution. Indeed, among the pleiotropic effects of statins could be included the alterations in membrane biophysical properties and their direct interaction with lipid rafts. Lipid rafts are highly dynamic, functional membrane microdomains that play an essential role for the life of the cells since they act as concentrating platforms for individual receptors that are activated by ligand binding [5]. Cellular responses governed by lipid rafts regulate both physiological and pathological processes, and the effectiveness of therapeutic strategies targeting pathological lipid rafts relies on the ability of an agent to selectively identify and disrupt these aberrant domains while preserving normal raft-dependent cellular functions [6]. Cancer cells typically exhibit elevated levels of membrane cholesterol and an increased abundance of lipid rafts compared with their non-tumorigenic counterparts. Numerous signaling pathways implicated in tumor development, such as the insulin-like growth factor system or phosphatidylinositol 3-kinase/AKT signaling, are strongly modulated by lipid rafts [7]. Furthermore, apoptosis induced by Fas/CD95 death receptor is mediated by the formation of Fas/CD95 aggregates in lipid rafts and this clustering can be achieved not only by interaction with its natural ligand but using also specific molecules such edelfosine. Edelfosine was the first antitumor drug reported to promote an apoptotic response via triggering ligand-independent activation of the Fas/CD95 death receptor, particularly effective in cancer cells derived from blood malignancies [8,9]. It is well established that lipophilic statins preferentially incorporate into cholesterol-rich membrane domains, thereby modifying membrane curvature and potentially impairing the ability of lipid rafts to recruit and cluster signaling proteins that depend on a defined membrane order [10,11]. A major limitation in delineating the direct effects of statins on lipid rafts is the overlap with their primary mechanism of action, the HMG-CoA reductase inhibition. A reduction in cholesterol levels alone is sufficient to destabilize lipid rafts, making it challenging to determine whether the observed alterations in these membrane microdomains arise from direct statin–membrane interactions or are simply secondary to cholesterol depletion. Model cell membranes are experimental systems that mimic the lipid organization of biological membranes, providing a platform to differentiate between the direct membrane effects of statins and their indirect, cholesterol-lowering properties. Lipid domains in model membranes can be investigated not only through fluorescence microscopy with dyes that preferentially partition into liquid-disordered (Ld) or liquid-ordered (Lo) phases, but also using advanced techniques such as Fluorescence Resonance Energy Transfer (FRET), super-resolution microscopy (SRM), and atomic force microscopy (AFM) [12]. However, artificial model membranes do not fully capture the complexity of biological membranes, particularly the dynamic interactions between proteins and lipids that play a critical role in membrane structure, signaling and transport. Considering the various challenges and concerns associated with statin repurposing, this manuscript seeks to address the following question: can statins be more broadly viewed as pharmacological agents that modulate membrane structure and function in diseases such as cancer, where aberrant membrane organization contributes to pathology?

## 2. The Fundamental Principle Behind the Use of Membrane Lipid Therapy in Cancer

Differences in membrane lipid composition between healthy and cancerous cells have driven the development of new therapeutic strategies aimed at targeting the cancer cell membrane to increase chemosensitivity and overcome multidrug resistance (MDR). For instance, phosphatidylserine (PS) and phosphatidylethanolamine (PE) which, under physiological conditions are present mainly in the inner leaflet of cell membranes, have increased expression on the outer membrane of tumor cells [13]. These differences have driven the development of phosphatidylserine (PS)-targeting antibodies that bind to exposed PS within the tumor microenvironment and can activate immune cells [14]. Another promising strategy focuses on increasing the concentration of ceramide, a sphingolipid typically found at low levels in the membranes of both healthy and tumor cells. Increased ceramide membrane levels induce cytochrome c release from mitochondria and activate the apoptotic pathway [15]. In this context, several therapeutic approaches are under investigation including ceramidase inhibitors that increase the sensitivity of head and neck squamous cell carcinoma to radiotherapy and chemotherapy by elevating ceramide levels or the use of ceramide analogues with pro-apoptotic properties [16,17].

However, cancer cell lipid composition differs not only between malignancy types but also fluctuate in time depending on tumor state: cholesterol levels in cell membranes are typically elevated during the early stages of cancer, but decrease at later stages. This reduction in membrane cholesterol enhances membrane fluidity and plasticity, which helps tumor cells penetrate blood vessels [18]. Instead of focusing exclusively on cholesterol depletion or altering cholesterol and sphingolipid levels, membrane lipid therapy (MLT) emphasizes the modulation of the membrane bilayer’s physical properties, including fluidity, curvature and elasticity [19]. A promising candidate for MLT is the synthetic fatty acid 2-hydroxyoleic acid (2OHOA), a potent antitumor drug that binds to the membrane bilayer, altering its structure and microdomain properties [20]. Among the proposed mechanism of action, 2OHOA treatment selectively increases membrane sphingomyelin levels through activation of sphingomyelin synthase, resulting in an increase in Lo domains and consequent alterations in membrane fluidity, as also observed in various artificial membrane models [21,22,23]. More recently, Lou and colleagues observed that 2OHOA does not activate sphingomyelin synthase and suggested that cancer suppression could be related to the 2OHOA-induced phosphatidylcholine reduction [24]. Molecular dynamics studies have shown that the loss of lipid asymmetry observed in cancer cells is associated with reduced membrane permeability to chemotherapeutic drugs such as cisplatin [25]. Altering the lipid architecture of cellular membranes can also change the localization and function of membrane proteins, thereby influencing downstream signaling involved in apoptosis, cell proliferation and differentiation [26]. A representative example is the finding that KRAS activation and signaling are highly dependent on the phospholipid composition of the plasma membrane, as different lipids exhibit distinct affinities for the protein [27]. In particular, the activity of KRAS has been directly related to membrane regions rich in phosphatidylserine, which interact with a polybasic amino acid region in the C-terminal part of this protein [28]. *KRAS* is an oncogene encoding a small GTPase that mediates downstream signaling of growth factor receptors. *KRAS* mutations impair its GTPase activity, leading to constitutive signaling and uncontrolled proliferation, contributing, among the others to pancreatic ductal adenocarcinoma, colorectal cancer, non-small cell lung cancer and endometrial carcinoma [29,30]. These alterations in membrane lipids, which regulate the function and localization of various peripheral signaling proteins, have been termed “lipid switches” because they profoundly modify cellular processes such as proliferation or apoptosis, highlighting the effective potential of MLT [31].

### 2.1. Statins as Modulators of Membrane Properties

Statins, a well-known group of cholesterol-lowering agents, are capable of influencing membrane lipid organization and composition in multiple ways. Statin-mediated cholesterol depletion can alter lipid rafts integrity, leading to activation or deactivation of membrane receptors and their associated proteins, such as death receptors, protein kinases, or calcium channels [32]. However, incorporation of statins into lipid bilayers directly alters bilayer properties, such as the intrinsic curvature, thickness, and fluidity [33]. This membrane perturbation is frequently associated with antiproliferative effects, as increased membrane permeability can facilitate greater intracellular accumulation of chemotherapeutic agents [34]. Indeed, MDR in cancer cells is not solely driven by the overexpression of P-glycoprotein, an ATP-dependent efflux pump that extrudes chemotherapeutic agents, but also depends on elevated levels of cholesterol and glycosphingolipids within the plasma membrane of tumor cells [35]. The synergistic effects of statins observed with conventional chemotherapeutic agents on cell viability are also associated with increased apoptosis, suppression of DNA repair pathways, and induction of cell cycle arrest [36,37,38]. The anticancer properties of statins, both as standalone agents or in combination with other therapies, have attracted considerable attention in recent decades and have been comprehensively summarized in a few excellent reviews [2,3,39]. The role of statins as membrane-modifying agents has been clarified through a variety of biomimetic membrane systems, which have undergone substantial advances over the past decade. These include bilayers in the form of vesicles that according to size are classified in small unilamellar vesicles (SUVs), large unilamellar vesicles (LUVs) or giant unilamellar vesicles (GUVs) and planar lipid bilayers, which are divided, based on how they are attached to a solid substrate, into Supported Lipid Bilayers (SLBs) and Tethered Bilayer Lipid Membranes (tBLMs).

**Figure 1:**
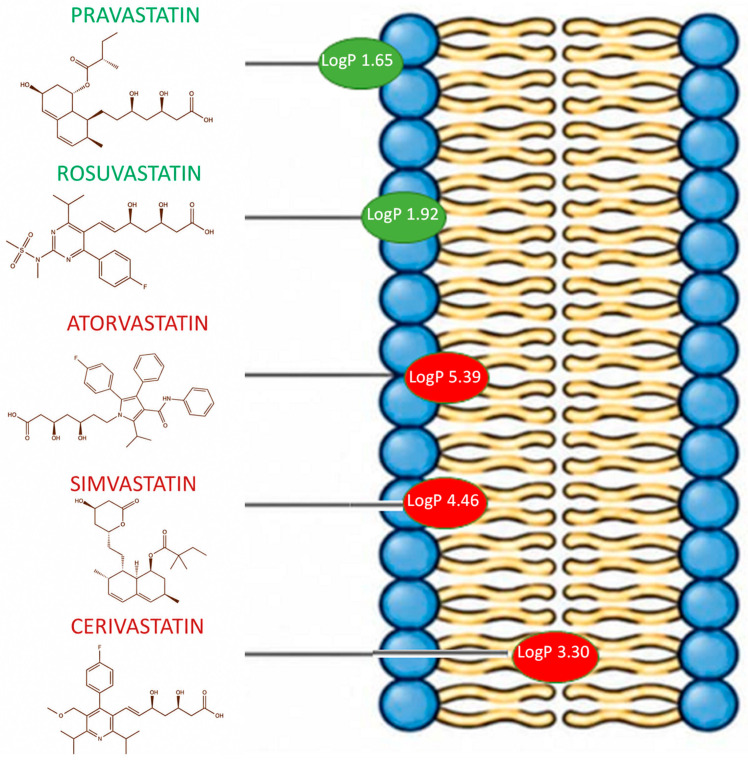
Different insertion of statins into lipid bilayer based on x-ray diffraction study [44]. Hydrophilic statins are colored in green while lipophilic ones in red. Lipophilicity influences statin`s deepness of insertion but the latter is not strictly connected with statin LogP since Cerivastatin LogP value of 3.30 is lower compared to simvastatin (4.46) or atorvastatin (5.39). Minimal chemical differences can influence drastically the interaction with the cellular membrane.

These models have enabled a deeper understanding of the molecular functions of the cell membrane as one of the major building blocks of life [40]. Insights on how statins interact with lipid membranes, partition into lipid phases and localize within cholesterol-rich domains, came from studies employing artificial membrane systems combined with the use of fluorescent dyes capable of reporting membrane order and lipid phase separation [34], The most common are lipophilic fluorescent probes that partition specifically into Lo or Ld phase allowing the imaging and study of membrane lateral heterogeneity [41]. A promising direction for the future is a better understanding of how statin lipophilicity influences their membrane distribution. LogP is a commonly used parameter for expressing statins lipophilicity. It is defined as the logarithm of the partition coefficient, which represents the ratio of the compound’s concentration in a nonpolar solvent (typically octanol) to its concentration in a polar solvent (usually water). In particular, lipophilic statins (higher LogP values) preferentially partition into ordered, cholesterol-rich regions of the plasma membrane, disrupting lateral lipid packing, inducing membrane thinning, and elevating local curvature stress, effects that induce destabilization of raft microdomains [42]. Hydrophilic statins (lower LogP values) may show greater affinity for sphingomyelin-enriched domains, specifically affecting the 2D crystalline structure [10]. These differences are important when considering the hypothesis that the biological effects of statins are influenced not only by their chemical properties but also by their specific positioning within cell membranes [43]. Notably, cerivastatin, withdrawn from the market in 2001 due to a high incidence of fatal rhabdomyolysis, was found to penetrate membranes more deeply than other statins, frequently reaching the central hydrocarbon core, while hydrophilic pravastatin and rosuvastatin were associated with the hydrated surface of the membrane [44] (Figure 1). Indeed, hydrophilic statins mainly rely on active transport mediated by members of the organic anion transporting polypeptides (OATPs) [45,46]. In several cancer types, altered expression, mis-localization, or functional impairment of these transporters can reduce statin accumulation within malignant cells, contributing to diminished therapeutic response and apparent statin resistance [47,48]. This is especially relevant in the adjuvant setting, where statins are being evaluated as complementary agents to chemotherapy and may partly explain the higher efficacy observed with lipophilic statins when used alone or in combination therapy [49,50,51]. Therefore, the lipophilicity of statins plays a crucial role in their pharmacokinetic and pharmacodynamic properties and is believed to contribute largely to their membrane-specific and, in general, cholesterol-independent effects [52,53]. Collectively these findings suggest that the potential membrane activity of statins is, at least in part, mediated by biophysical targeting mechanisms, the elucidation of which may provide important insights into the wide range of pleiotropic effects of statins and their influence on cellular function [54].

### 2.2. Overcoming Challenges in Statin Repurposing for MLT

Several concerns halt the enthusiasm for the clinical use of statins within the framework of MLT. First, it remains challenging to disentangle their direct effects on lipid membranes from the indirect consequences of cholesterol depletion. A commonly used way to separate direct statin–membrane effects from indirect cholesterol-mediated effects is to combine cholesterol rescue experiments with matched depletion controls. Cells can be treated with statins and compared against a matched condition where cholesterol is depleted by agents such as methyl-β-cyclodextrin, ensuring equivalent reductions in membrane cholesterol. A rescue condition is then introduced by replenishing cholesterol after statin treatment. If membrane changes observed under statins are reproduced by depletion alone and fully reversed by cholesterol repletion, the effects are primarily indirect via cholesterol inhibition; if statins still produce distinct changes in reconstituted systems or persist beyond cholesterol normalization, this supports direct statin–lipid membrane interactions. A key limitation of this approach is that matching total cholesterol levels does not ensure equivalent membrane organization. While cholesterol depletion with methyl-β-cyclodextrin is rapid and primarily affects the plasma membrane, statins alter cholesterol more gradually through inhibition of the mevalonate pathway, leading to broader changes in lipid distribution and cellular metabolism. As a result, observed differences may reflect altered trafficking, signaling, or lipid remodeling rather than a true direct effect related to statin–membrane interaction. Once again, complementary experiments in cell-free systems are strongly recommended to match the observation on biological samples.

**Figure 2:**
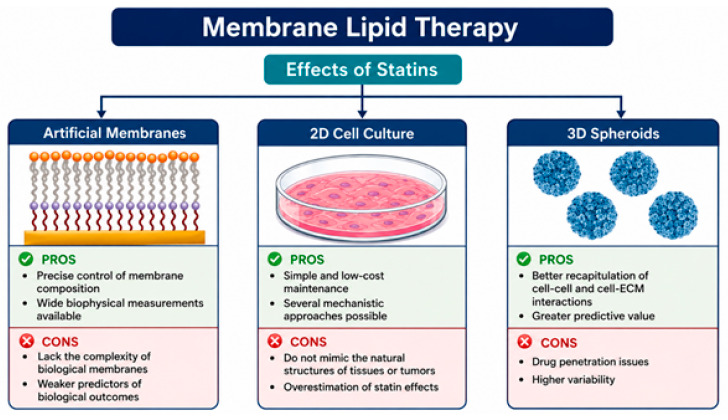
Different models used to investigate the role of statins in membrane lipid therapy. Artificial membranes, like the illustrated tethered bilayer lipid membranes, lack the complexity of biological systems and their interactions with intra- and extracellular components, but allow a straightforward evaluation of changes in biophysical membrane properties. In 2D cell culture models, distinguishing between the direct effects of statins on membrane lipids and the indirect effects arising from cholesterol depletion is often difficult, potentially leading to an overestimation of statins’ direct impact on membrane properties. 3D spheroids and organoids provide more physiologically relevant systems to assess the functional consequences of statin-induced membrane lipid remodeling, such as increased sensitivity to cytotoxic agents. However, drug penetration issues and greater variability are frequently observed.

Another pitfall relies on the fact that lipophilic statins, which more easily penetrate and modify the lipid bilayer, are frequently associated with adverse effects such as myopathy and rhabdomyolysis, although a genetic predisposition is also involved [55,56]. It is established that the risk of myopathy rises with increasing dose; therefore, conditions that impair statin metabolism or excretion may result in elevated circulating statin levels and a greater risk of myopathy [57]. Computational analyses focusing on the pharmacokinetic behavior, binding affinities, and structural stability of statin analogs can facilitate the identification of compounds that retain cholesterol-inhibiting activity while exhibiting reduced side effects [58]. Evidence indicates that amine-containing residues, especially cycloalkyl sulfonamide analogues, exhibit strong hepato-selectivity and potent *in vivo* inhibition of cholesterol synthesis [59,60]. Furthermore, a major limitation in our current understanding of statin–membrane interactions lies in the discrepancy between experimental and physiological conditions. Therapeutically relevant plasma concentrations of statins are typically in the nanomolar range, whereas many mechanistic studies employ micromolar concentrations, raising doubts about the translational relevance of *in vitro* observations [61,62]. In light of these limitations, the functional consequences of statin-induced membrane lipid remodeling should be interpreted with caution, with the aim of validating this mechanistic promise in models that better reflect clinical reality, such as 3D culture systems including spheroids (Figure 2). A recent study comparing the *in vitro* effects of statins in 2D and 3D cancer models showed that statins reducing cell viability in monolayer culture also tend to suppress spheroid formation and growth [63]. Comparisons between 2D and 3D models do not always produce consistent results. Although statin effects appear similar in 2D and 3D pancreatic cancer models (BxPC-3, MIA PaCa-2, and PANC-1), they differ in breast cancer models (MCF-7 and MDA-MB-231), indicating that spheroids introduce structural complexity more representative of *in vivo* tissues and create heterogeneous lipid environments across outer and inner regions [64,65]. Among statins, atorvastatin has exhibited marked anti-angiogenic and pro-apoptotic activity in glioma spheroid models, characterized by reduced VEGF expression and enhanced caspase-3 levels [66]. This observation is further supported by animal studies showing that atorvastatin increases the anticancer effect of temozolomide through Ras and ERK-related mechanisms to inhibit glioblastoma growth [67]. In a biobank of 260 patient-derived pancreatic cancer organoids, statins effectively targeted chemo-resistant Pancreatic Ductal Adenocarcinoma (PDAC) models [68]. Statin treatment reduced protein glycosylation, cholesterol levels, and epithelial-to-mesenchymal transition-associated signatures, all features linked to therapy resistance. These findings suggest that statins may help overcome chemoresistance in PDAC. Importantly, a phase II clinical trial combining atorvastatin with chemotherapy demonstrated promising clinical responses in patients with advanced pancreatic cancer, supporting the translational potential of statin-based combination therapies [68]. Although statins have demonstrated promising anticancer effects in spheroid and organoid models, the majority of studies have evaluated conventional oncological endpoints rather than directly examining changes in membrane lipid organization. Moreover, the implications of the frequently reported spheroid disintegration remain poorly understood. Further studies are therefore needed to determine whether this effect contributes to therapeutic efficacy or, conversely, could increase the risk of tumor cell dissemination and metastatic progression. Observations of statin-induced membrane remodeling *in vivo* are comparatively limited, as the direct actions of statins on membrane structure and organization are often difficult to decouple from their broader systemic and cellular effect. In one study, statins alter the equilibrium of transbilayer cholesterol distribution in mice synaptosomal plasma membranes, thereby affecting cholesterol accessibility to distinct cellular domains and consequently modifying the distribution and organization of lipid rafts [69]. This observation is particularly relevant given the critical role of membrane cholesterol redistribution in driving the amyloidogenic processing of the Amyloid Precursor Protein (APP) during Alzheimer’s disease, as demonstrated in another animal study, using APP751_SL_ mice [70]. Simvastatin treatment increased the levels of insoluble Aβ1–40 and Aβ1–42 but reduced the levels of soluble Aβ1–40 in the brain.

In the context of clinical trials, a randomized controlled study by Coccia and colleagues demonstrated that preoperative simvastatin therapy preserved erythrocyte membrane fluidity during the oxidative stress associated with cardiopulmonary bypass [71]. Simvastatin-treated patients exhibited reduced lipid peroxidation and improved membrane functional properties compared with controls. Similarly, rosuvastatin treatment increases the fluidity of the erythrocyte membrane in patients with coronary artery disease, supporting the hypothesis that statins can modulate *in vivo* membrane biophysics beyond their cholesterol-lowering effects [72].

## 3. Conclusions

Although both biological and model membranes have provided valuable mechanistic insights into how statins can alter membrane lipid properties, the application of statins in MLT still faces several important limitations, including dose and clinical feasibility concerns or the absence of a clear distinction between direct effects on lipid membranes and indirect effects resulting from cholesterol depletion. Filling this translational gap requires integrating mechanistic insight with clinically relevant dosing in appropriate experimental models.

## Data Availability

No new data were created or analyzed in this study. Data sharing is not applicable to this article.
